# Supplementary material for: Pentacyclic Triterpenoids from Sabia discolor Dunn and Their α-Glycosidase Inhibitory Activities
Source: Molecules. 2022 Mar 27;27(7):2161. doi: 10.3390/molecules27072161 (PMC9000864; doi:10.3390/molecules27072161)
Supplement: Supplementary file 1 [file molecules-27-02161-s001.zip › molecules-1632378-supplementary.pdf]

Supporting Information

Pentacyclic triterpenoids from *Sabia discolor* Dunn and their *α*-glycosidase inhibitory activities

Jin-Hong Ma[1, 2, 3], Dan Hu[1, 2], Lu-Lu Deng[1, 2], Jiang Li[1, 2], Xiao-Jiang Hao[1, 2, 4], Shu-Zhen Mu[1, 2*]

1 State Key Laboratory of Functions and Applications of Medicinal Plants, Guizhou Medical University, Guiyang 550014, China;
jinhongma2022@163.com (J.H.M.); danhu1996@163.com (D.H.); luludeng@gzcnp.cn (L.L.D.) jiangli@gzcnp.cn (J.L.); haoxj@mail.kib.ac.cn (X.J.H.)

2 The Key Laboratory of Chemistry for Natural Products of Guizhou Province and Chinese Academy of Sciences, Guiyang 550014, China

3 School of Pharmacy, Guizhou Medical University, Guiyang 550025, China

4 Kunming Institute of Botany, Chinese Academy of Sciences (CAS), Kunming 650201, China

* Correspondence: muzi0558@126.com

1D, 2D NMR, HRMS and IR spectra data of compounds 1, 4, 7, 8 and 9

Compound 1

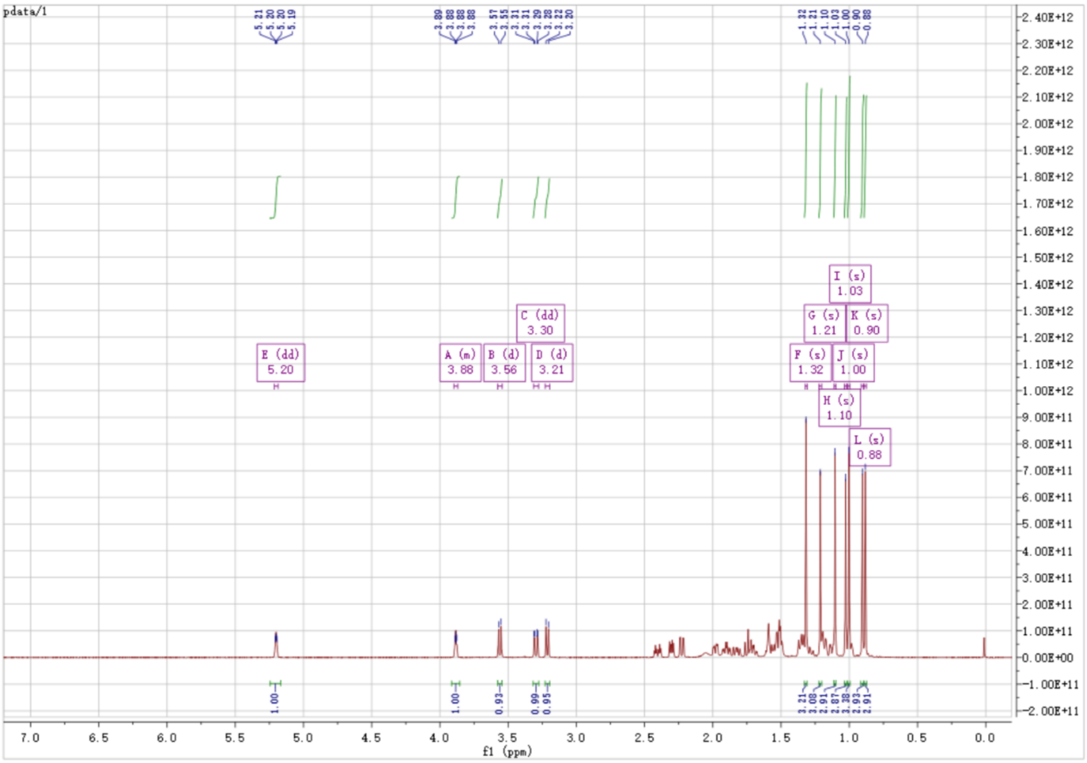

Figure S1.[1] H NMR spectrum of compound **1** in $CDCl_3$ (600 MHz).

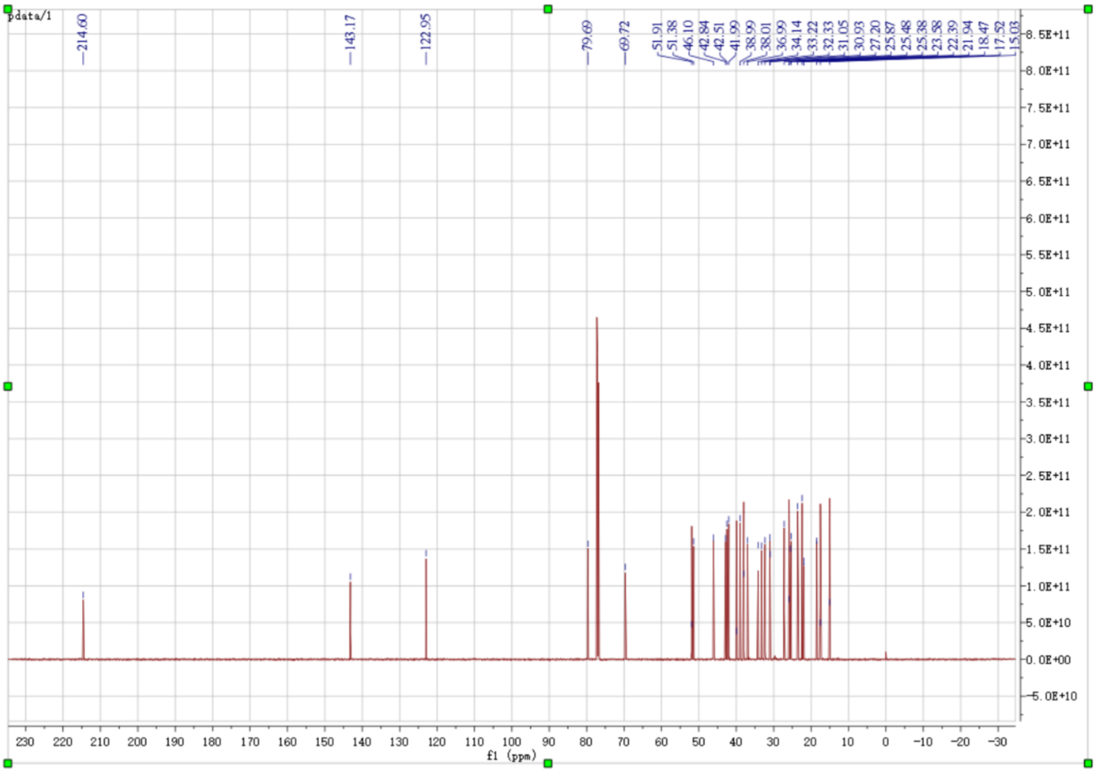

Figure S2. [13] C NMR spectrum of compound **1** in $CDCl_3$ (150 MHz).

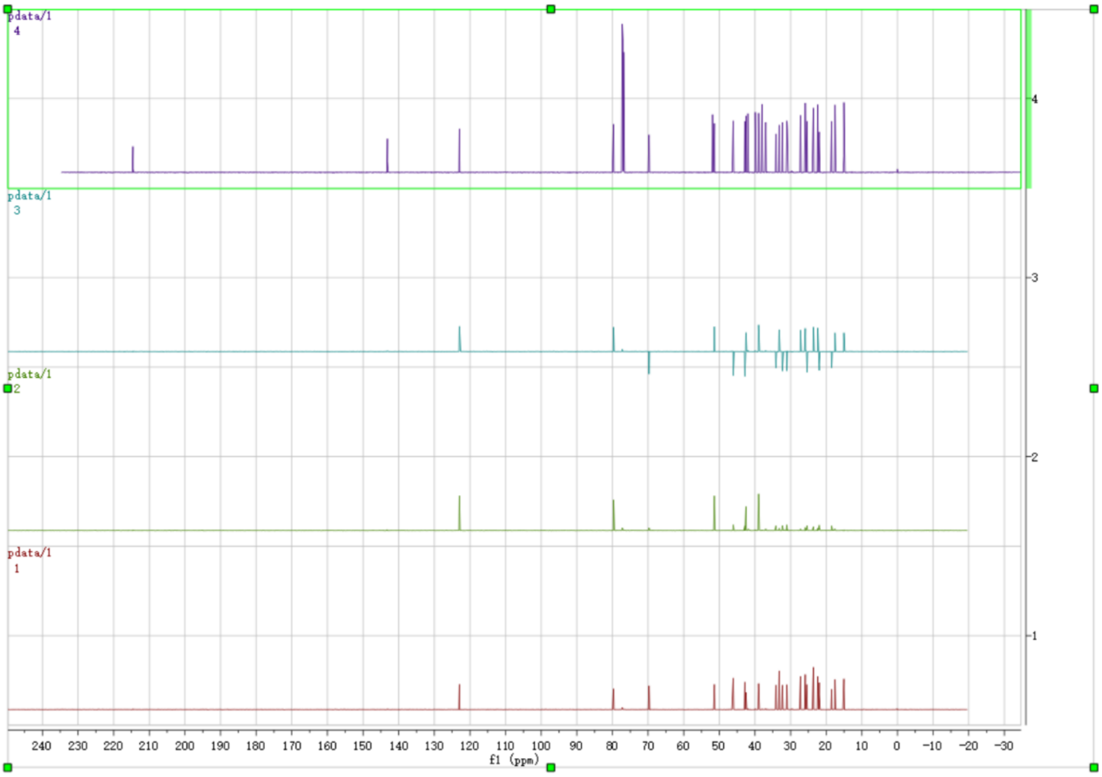

Figure S3. DEPT of compound **1** in $CDCl_3$ (150 MHz)

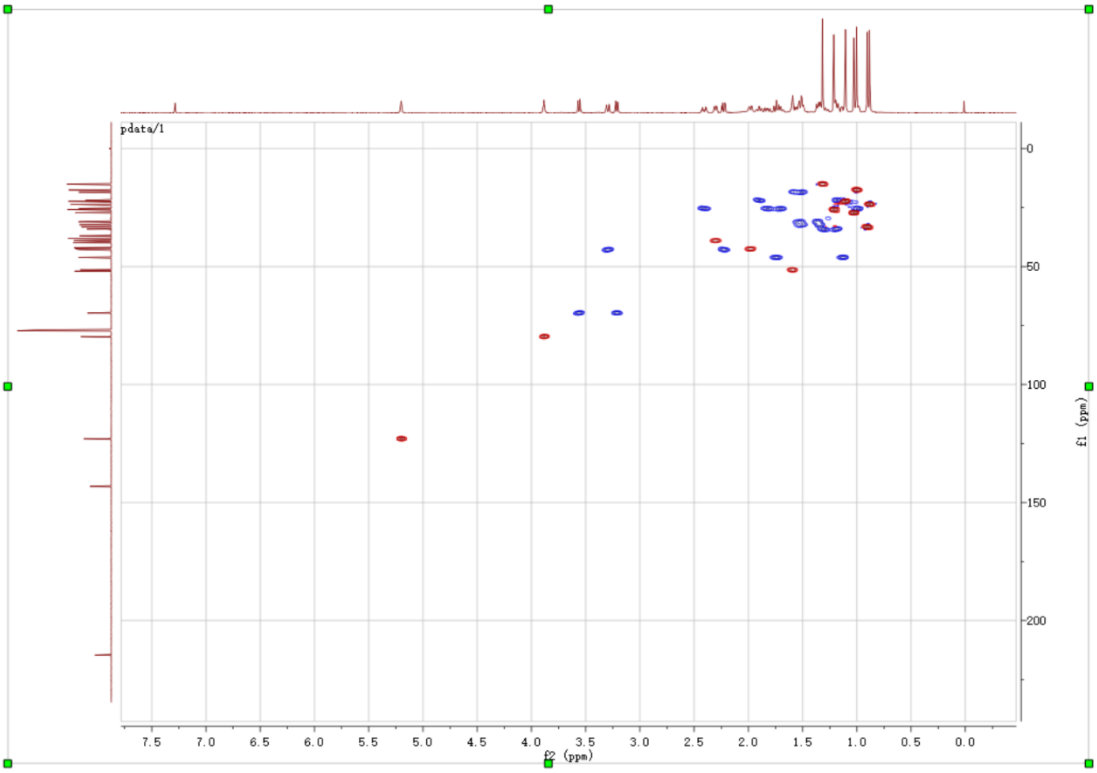

Figure S4. HSQC spectrum of compound **1** in $CDCl_3$

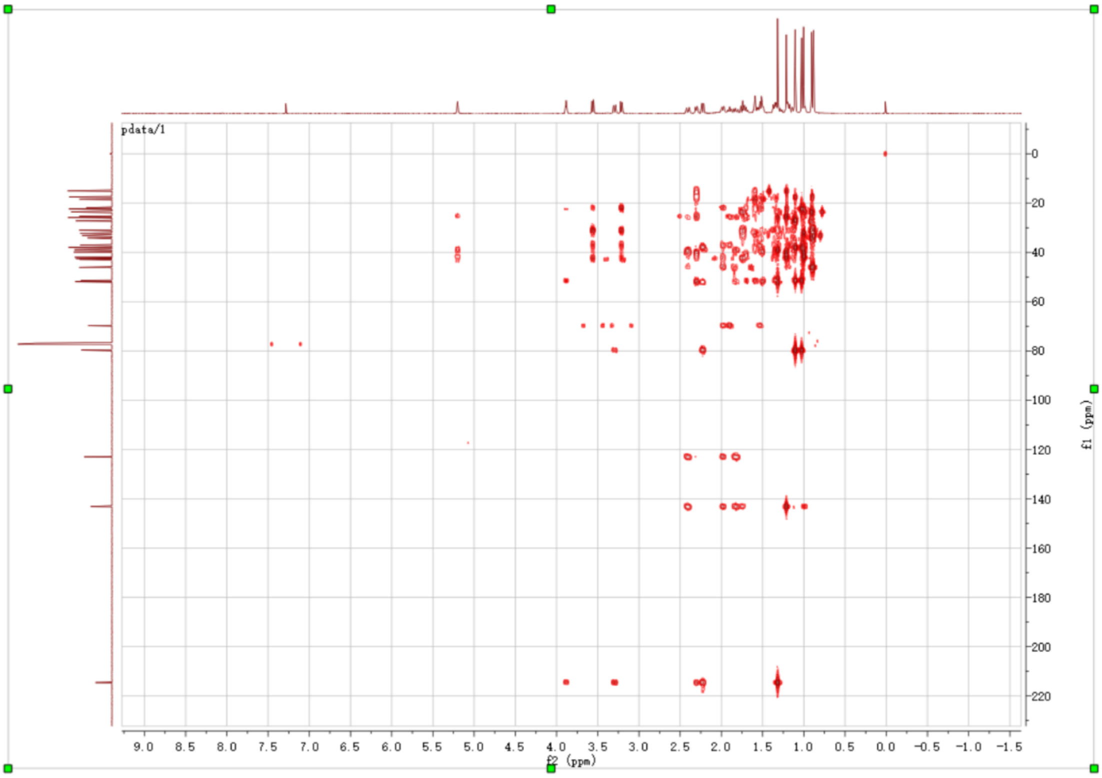

Figure S5. HMBC spectrum of compound **1** in $CDCl_3$

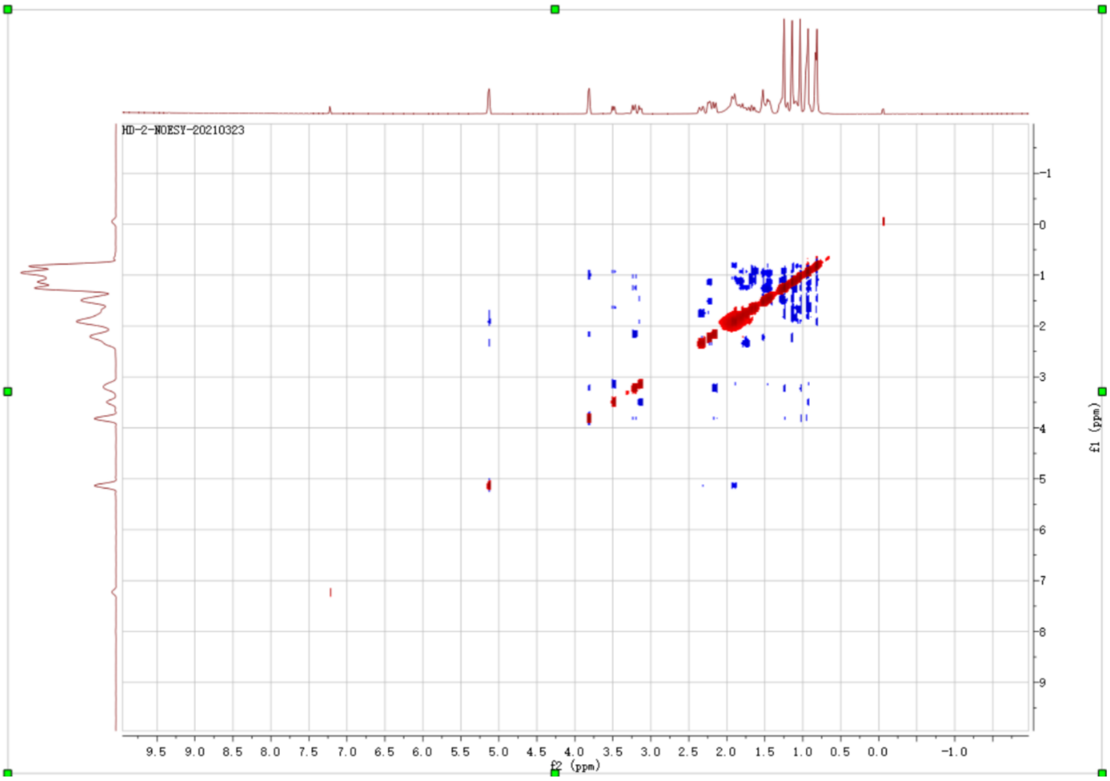

Figure S6. NOESY spectrum of compound **1** in $CDCl_3$.

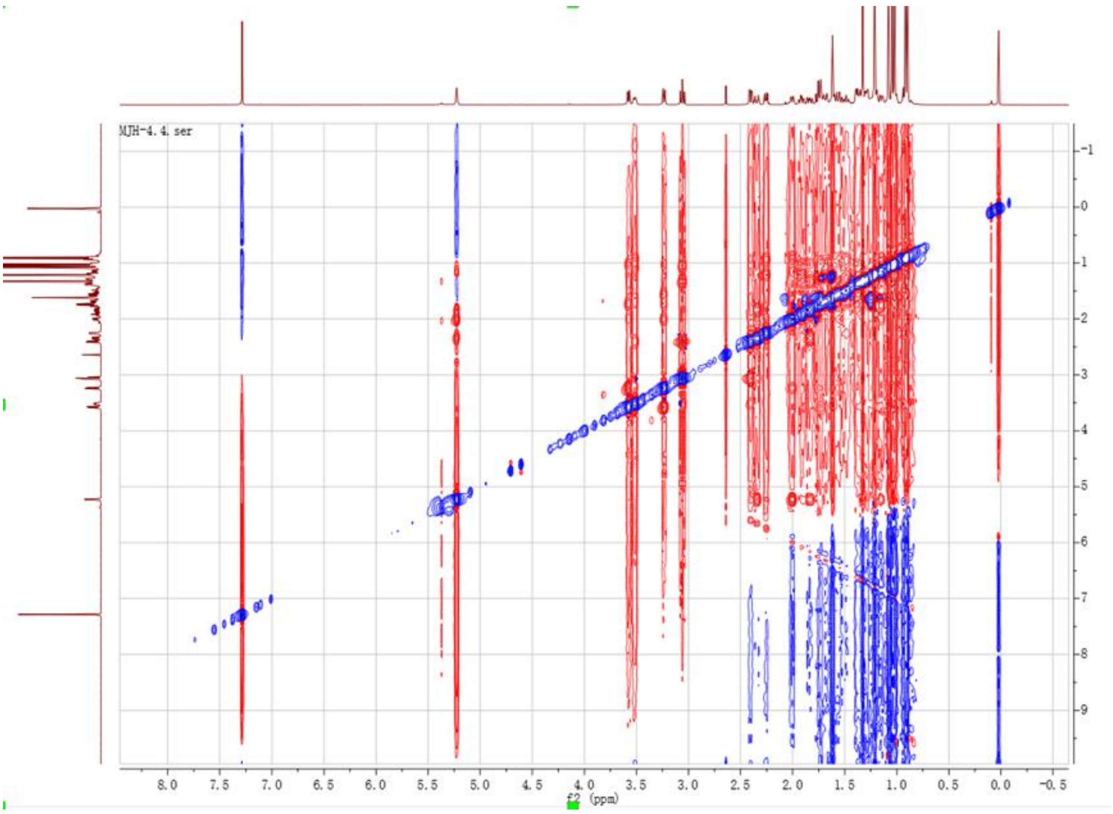

Figure S7. NOESY spectrum of compound **4** in $CDCl_3$.

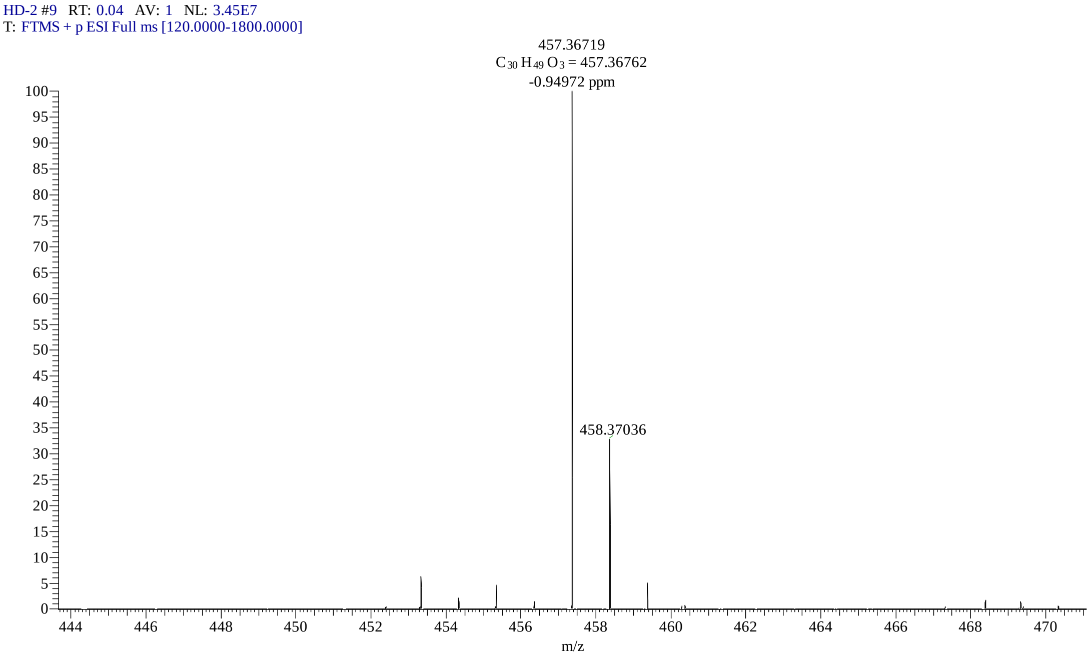

Figure S8. HRMS of compound **1** in $CDCl_3$

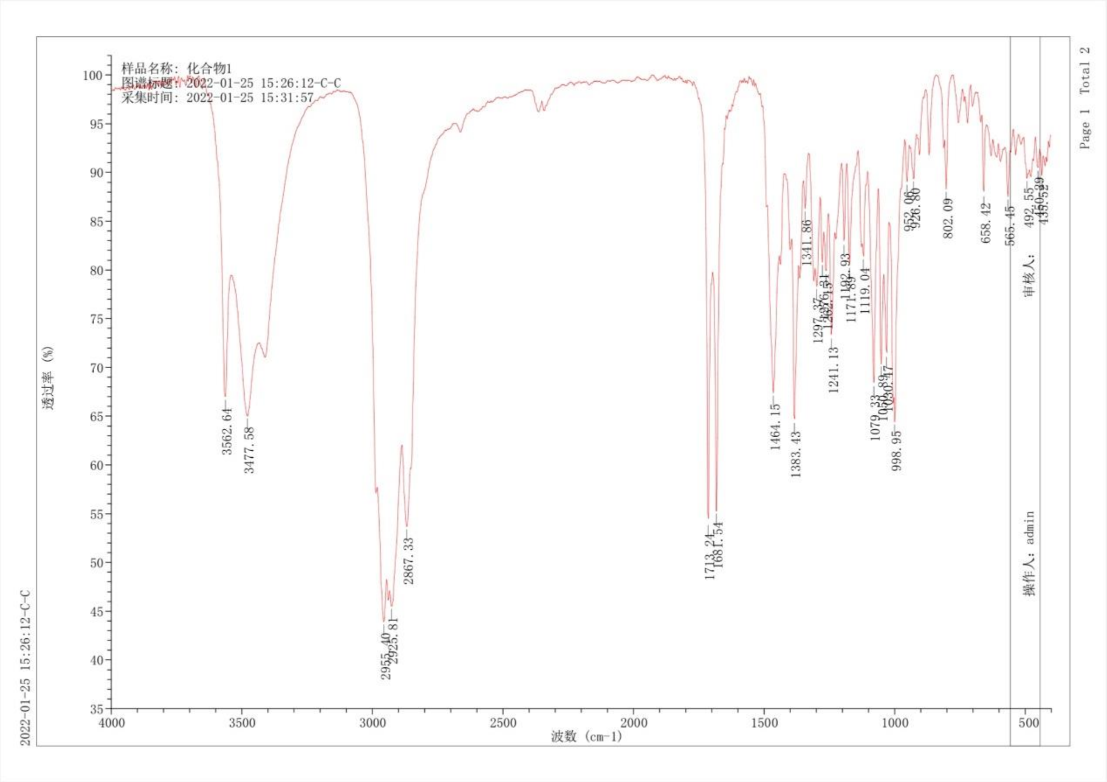

Figure S9. IR spectrum of compound **1**

波数 (cm-1)	%T
435.52	89.71
450.29	90.47
492.55	89.39
565.45	87.49
658.42	87.83
802.09	88.29
926.80	89.33
952.06	89.01
998.95	64.40
1030.47	71.36
1050.89	70.36
1079.33	68.33
1119.04	81.36
1171.89	80.47
1192.93	82.85
1241.13	73.23
1262.15	79.80
1276.31	80.79
1297.37	78.31
1341.86	86.28
1383.43	64.56
1464.15	67.37
1681.54	55.26
1713.24	54.09
2867.33	53.68
2925.81	45.51
2955.40	43.89
3477.58	65.03
3562.64	66.94

Figure S10. IR data of compound **1**

Compound **7**

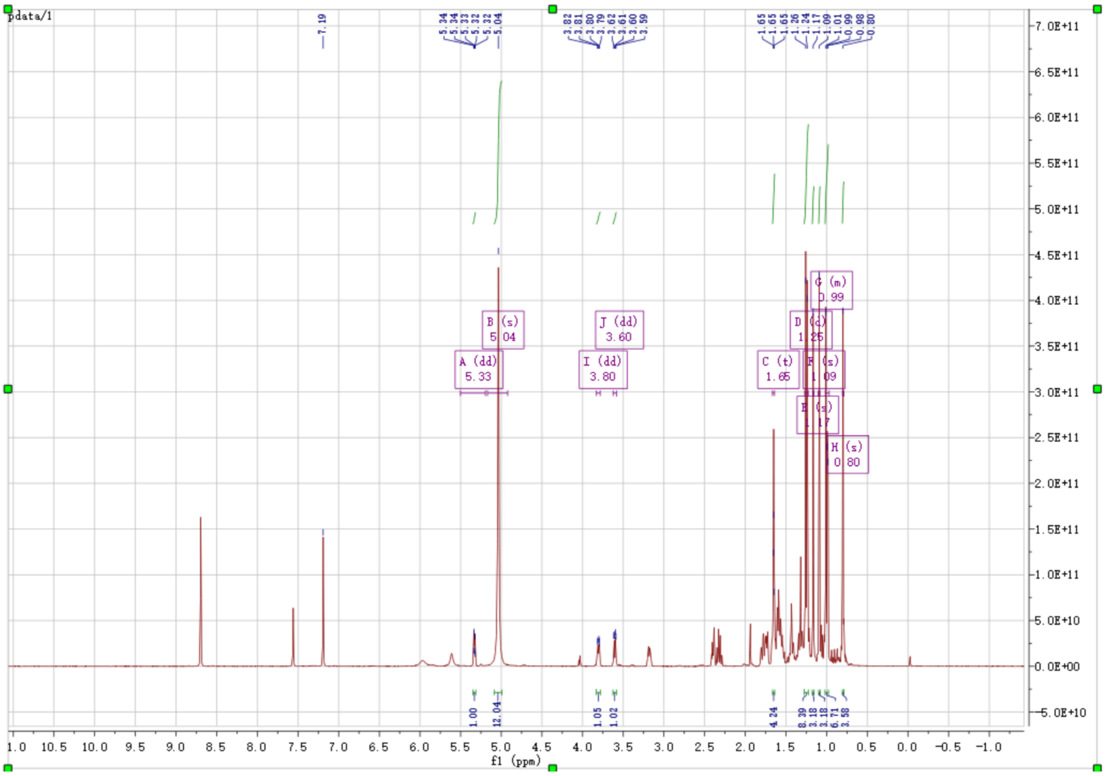

Figure S11.¹H NMR spectrum of compound **7** in C_5D_5N (600 MHz)

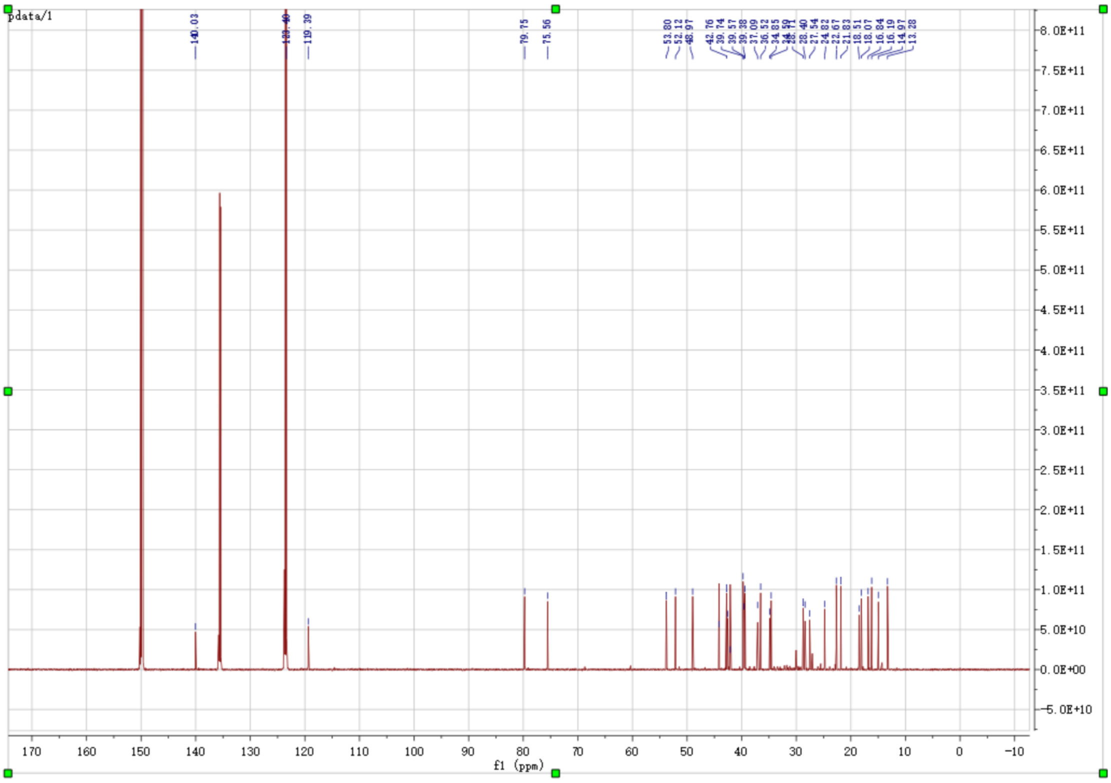

Figure S12. ¹³C NMR spectrum of compound **7** in C_5D_5N (150 MHz)

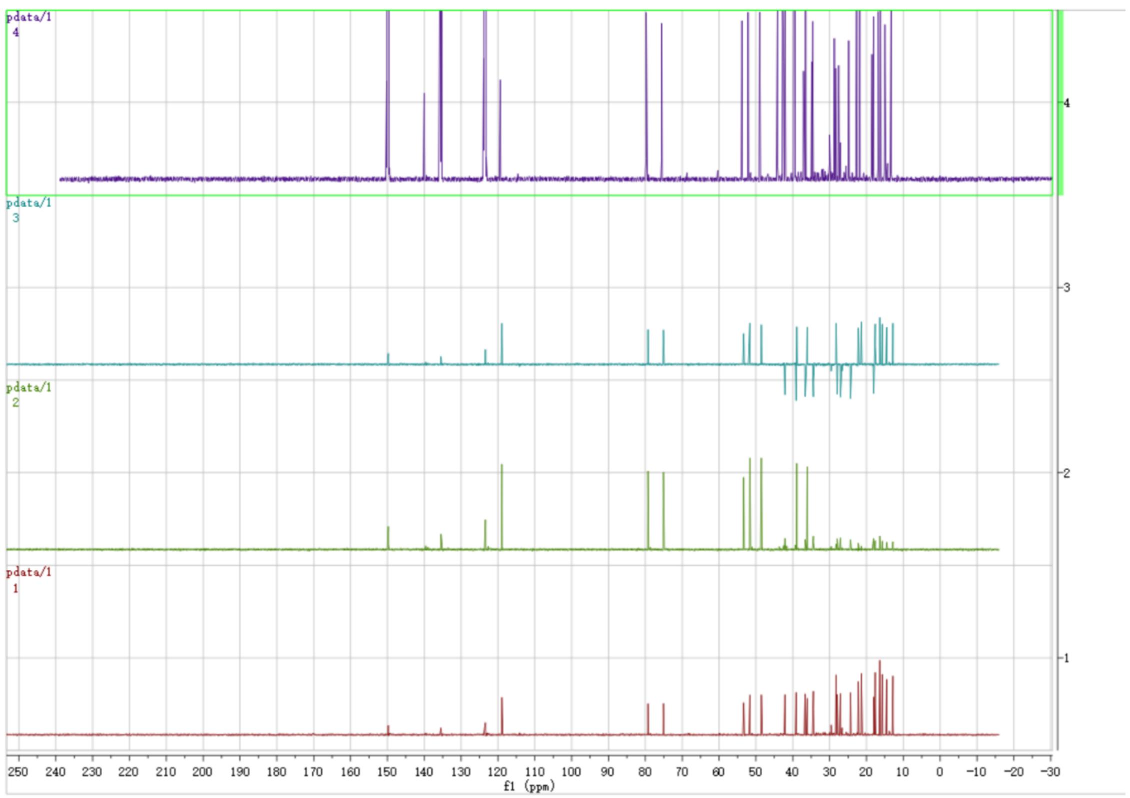

Figure S13. DEPT of compound **7** in C_5D_5N (150 MHz)

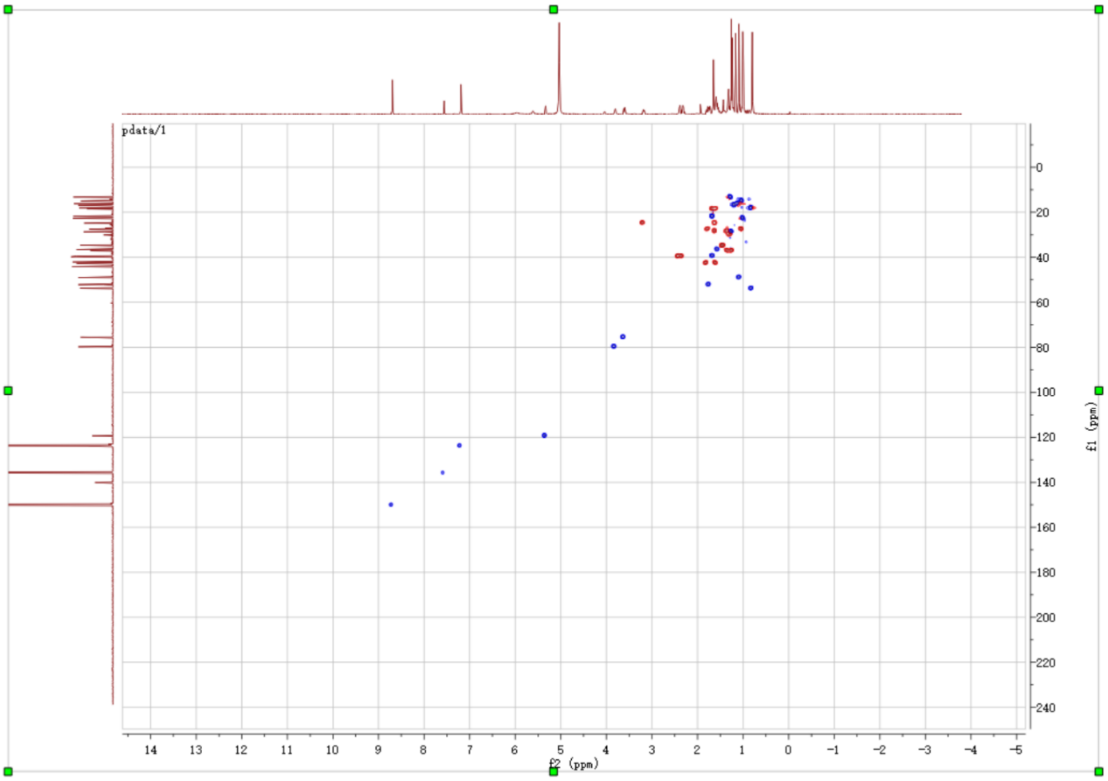

Figure S14. HSQC spectrum of compound **7** in C_5D_5N

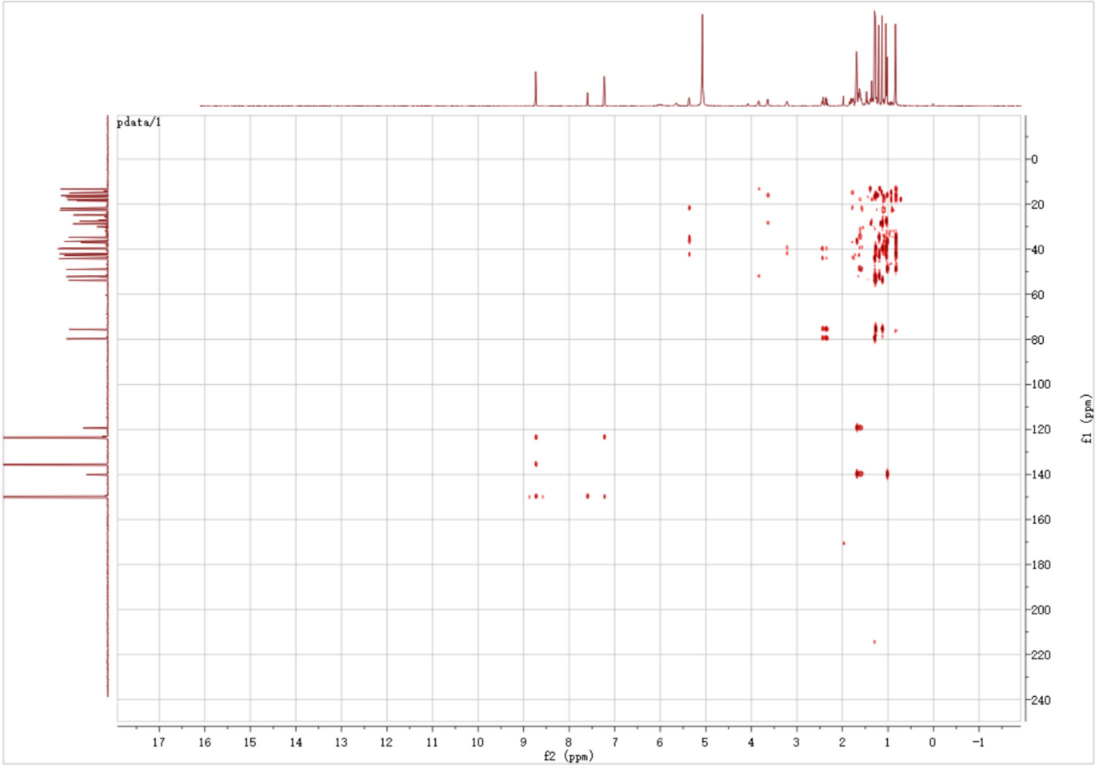

Figure S15. HMBC spectrum of compound **7** in C_5D_5N

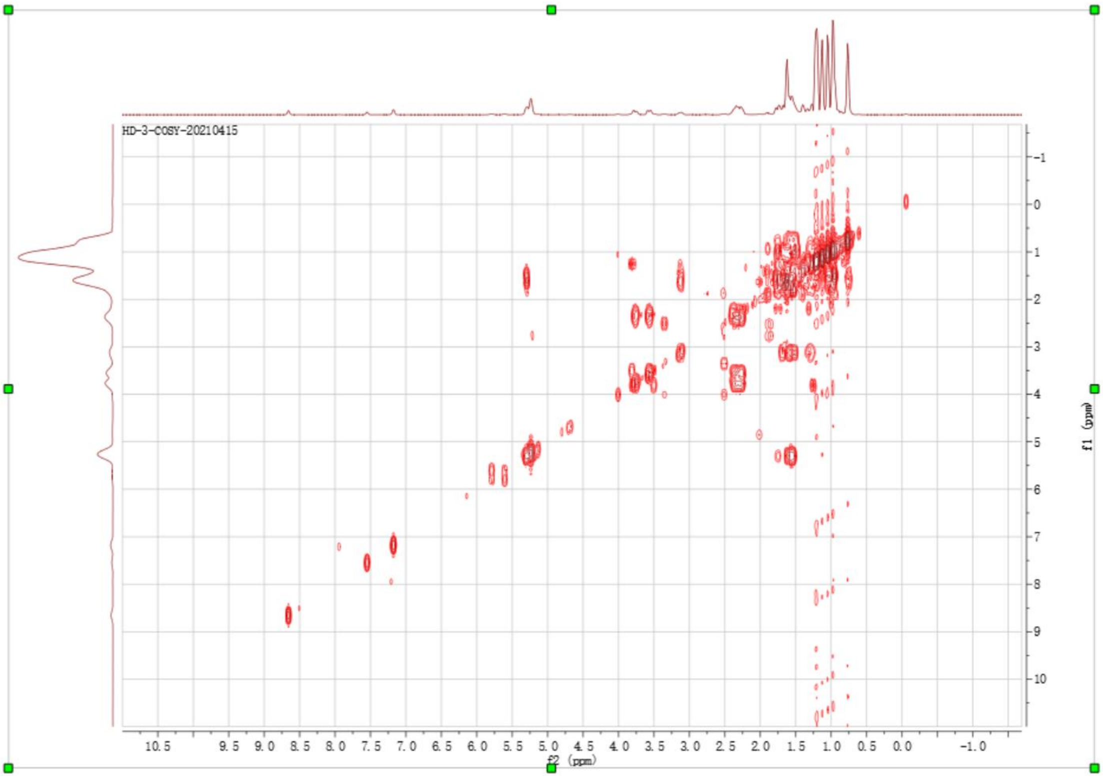

Figure S16. ^{1}H-^{1}H COSY spectrum of compound **7** in C_5D_5N

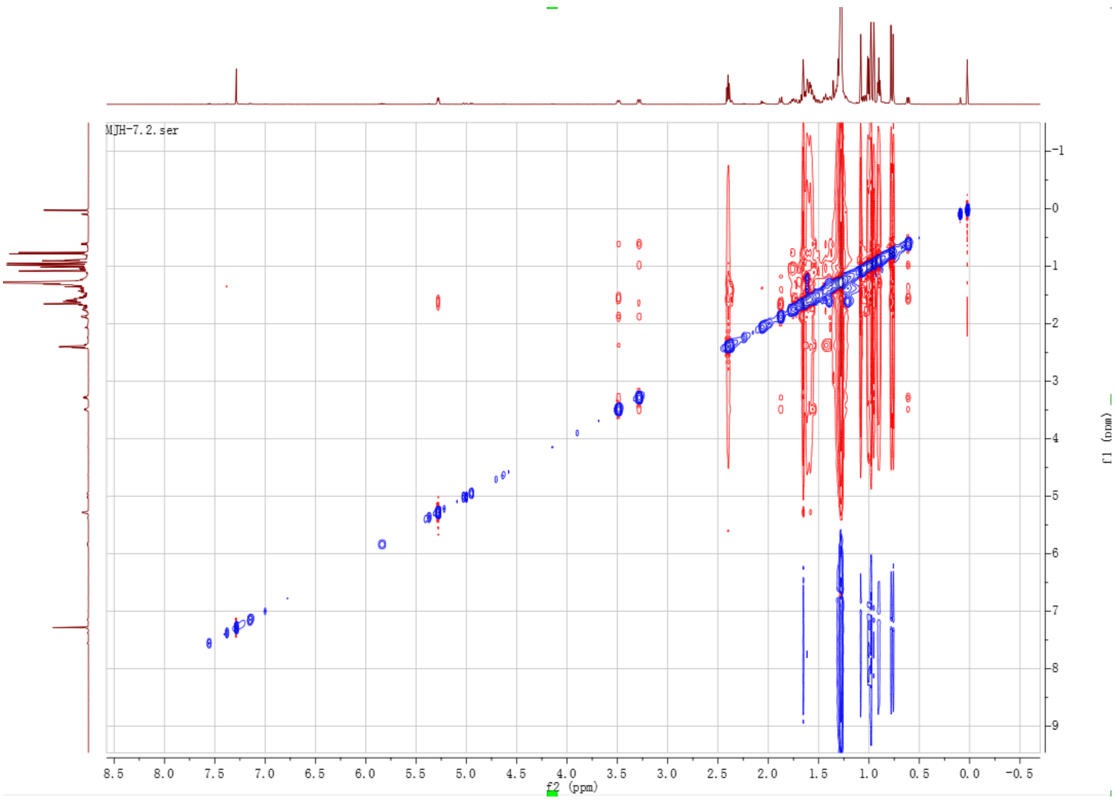

Figure S17. NOESY spectrum of compound **7** in C_5D_5N.

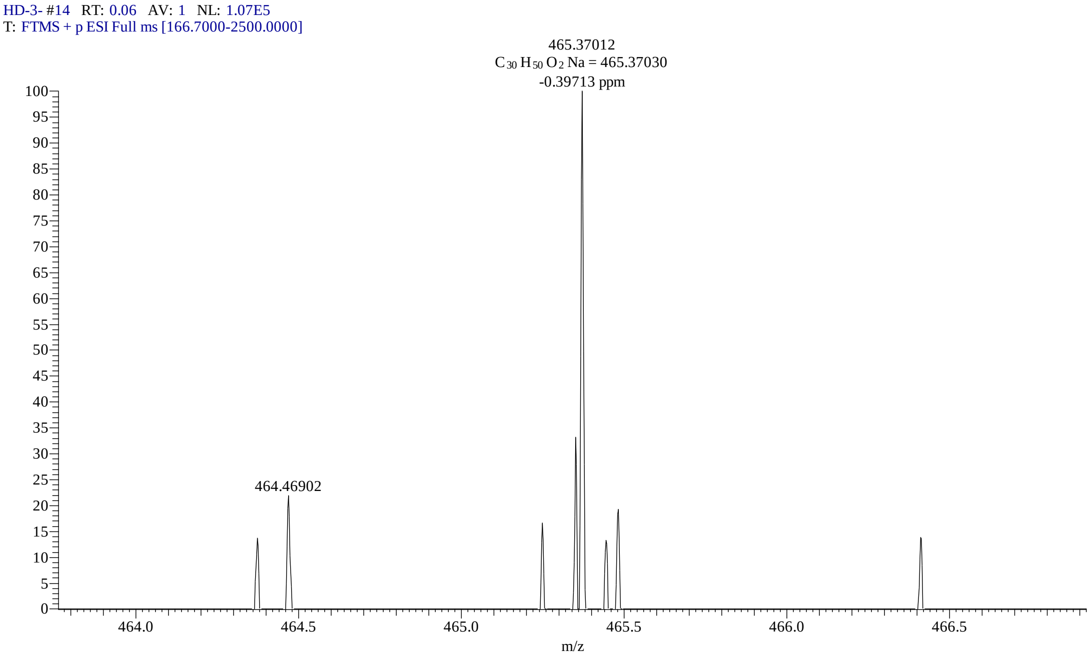

Figure S18. HRMS spectrum of compound **7** in C_5D_5N

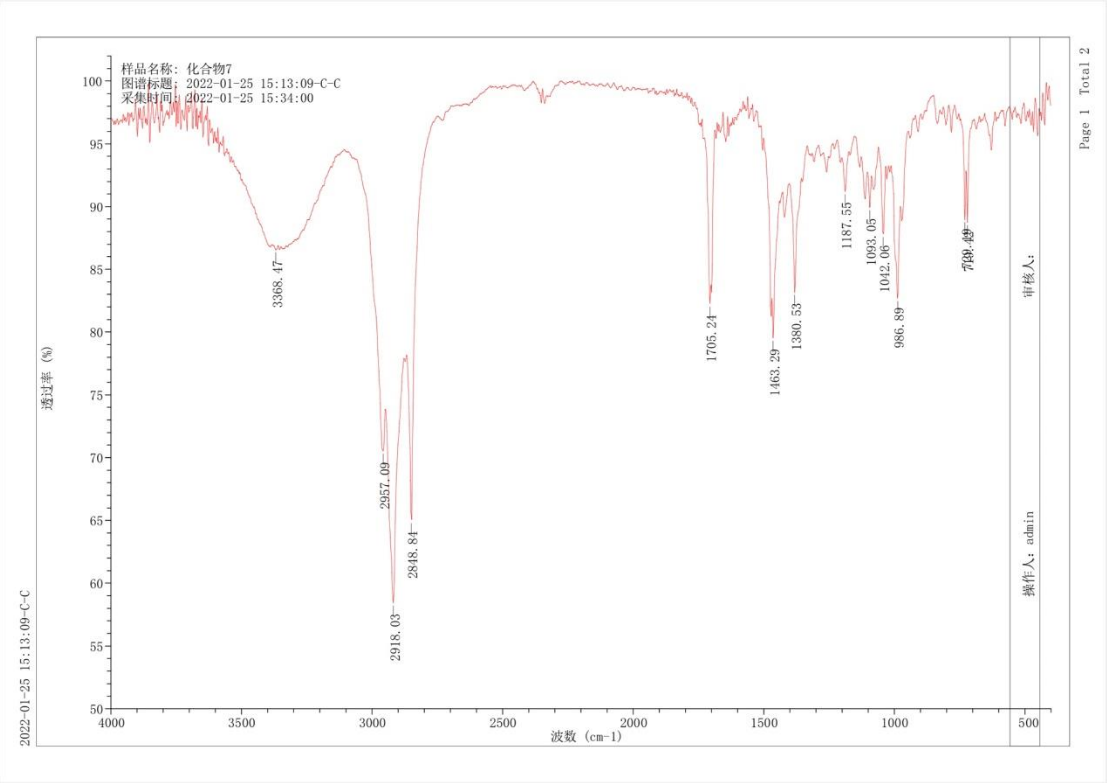

Figure S19. IR spectrum of compound **7**

波数 (cm-1)	%T
719.42	88.72
729.19	88.96
986.89	82.66
1042.06	87.79
1093.05	89.93
1187.55	91.19
1380.53	83.17
1463.29	79.51
1705.24	82.22
2848.84	65.00
2918.03	58.42
2957.09	70.52
3368.47	86.56

Figure S20. IR data of compound **7**

Compound **8**

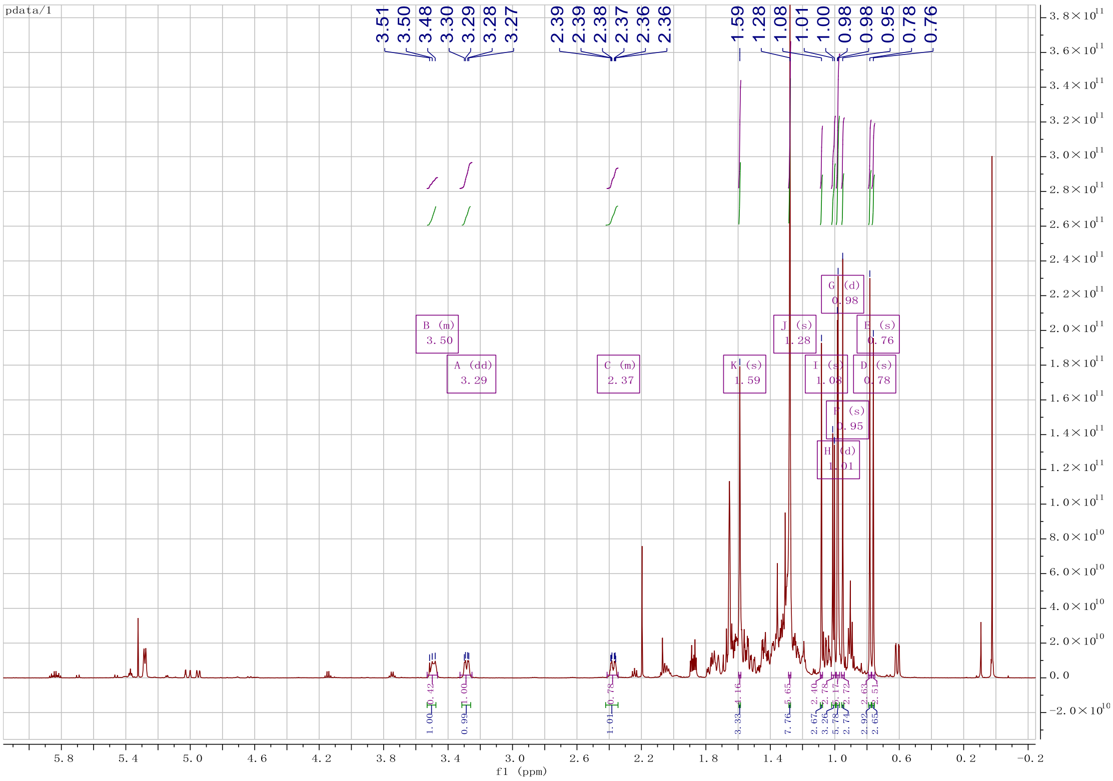

Figure S21. ^{1}H NMR spectrum of compound **8** in $CDCl_3$ (600 MHz)

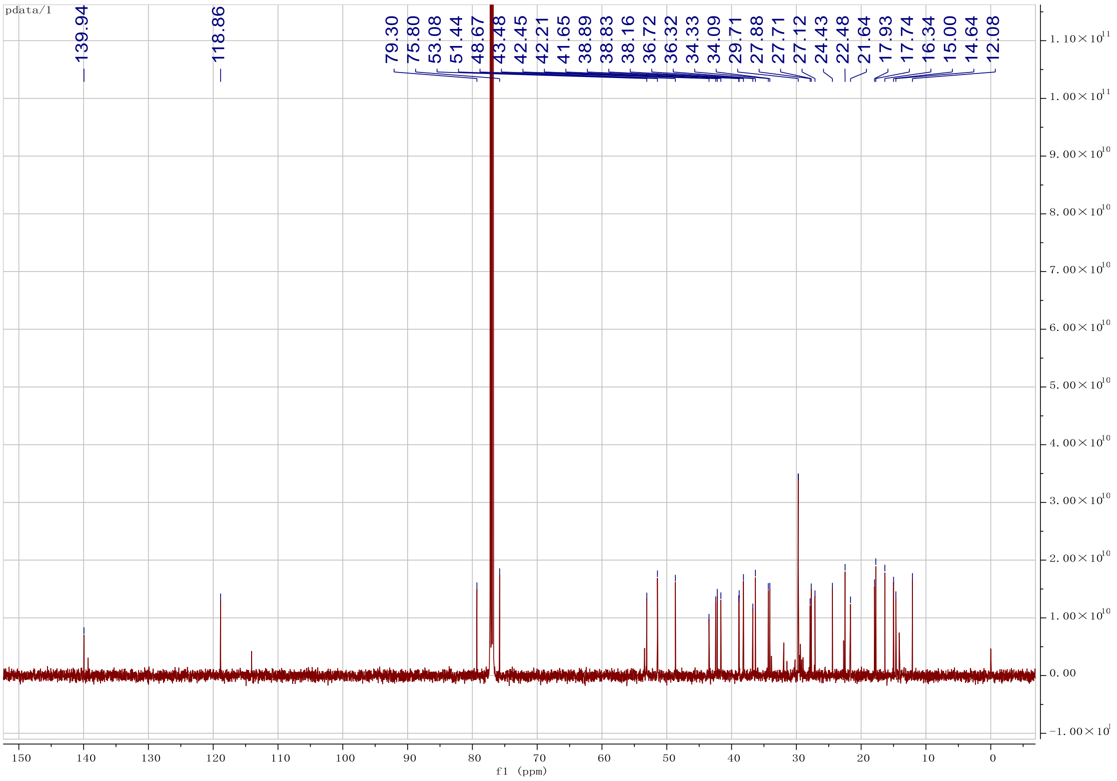

Figure S22. ^{13}C NMR spectrum of compound **8** in $CDCl_3$ (150 MHz)

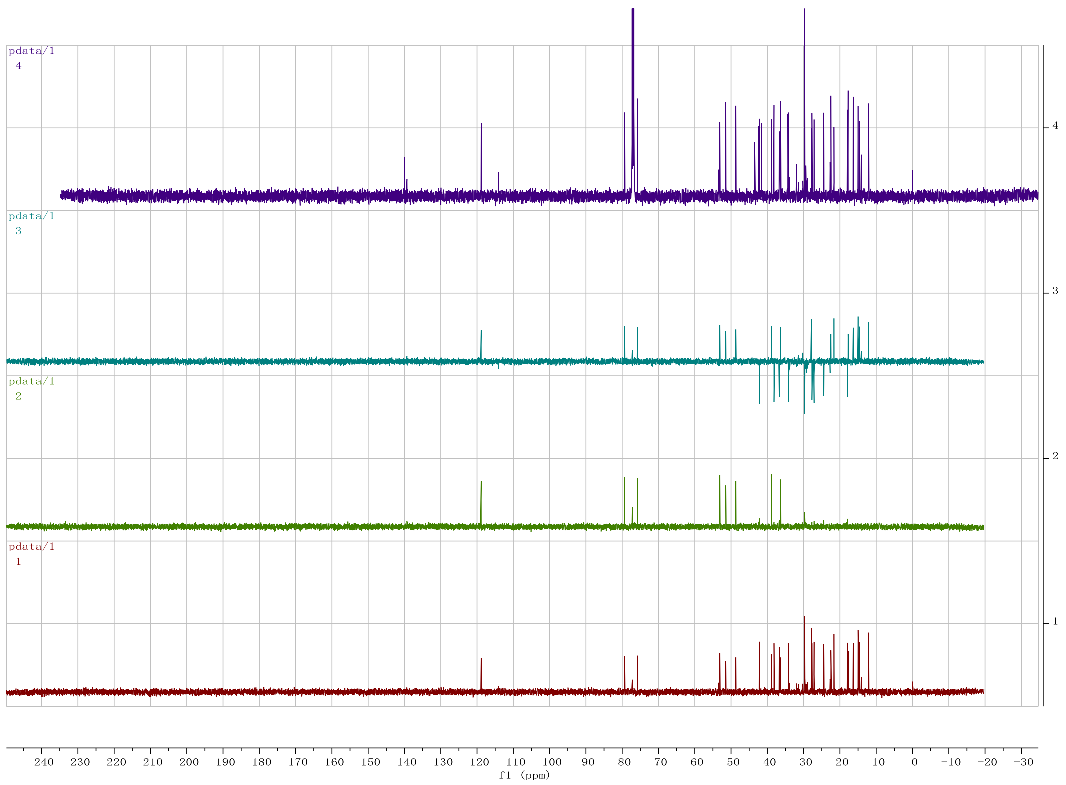

Figure S23. DEPT of compound **8** in $CDCl_3$ (150 MHz)

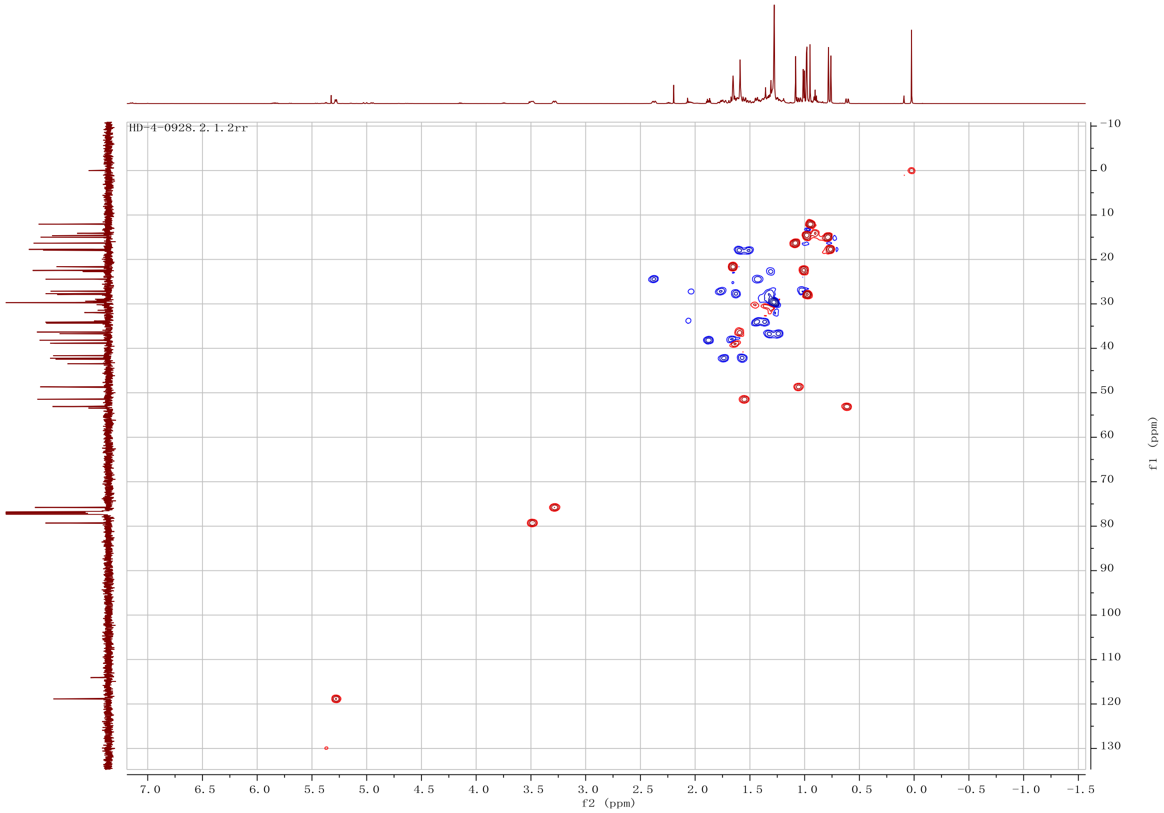

Figure S24. HSQC spectrum of compound **8** in $CDCl_3$

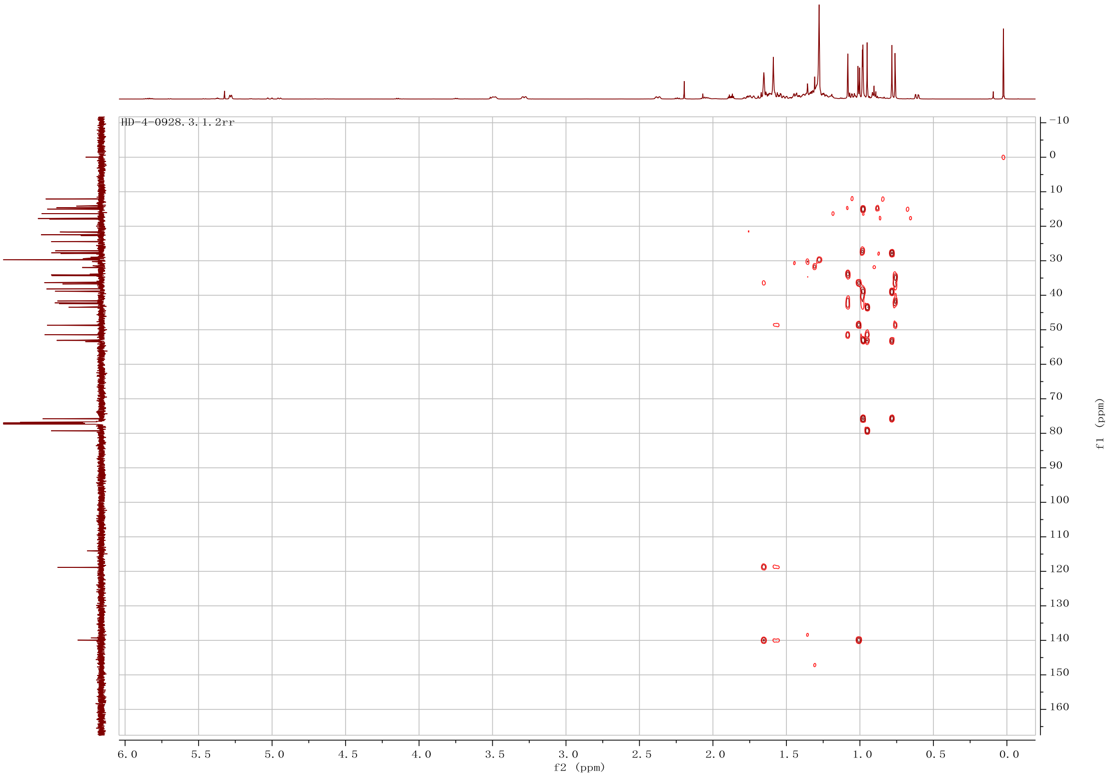

Figure S25. HMBC spectrum of compound **8** in $CDCl_3$

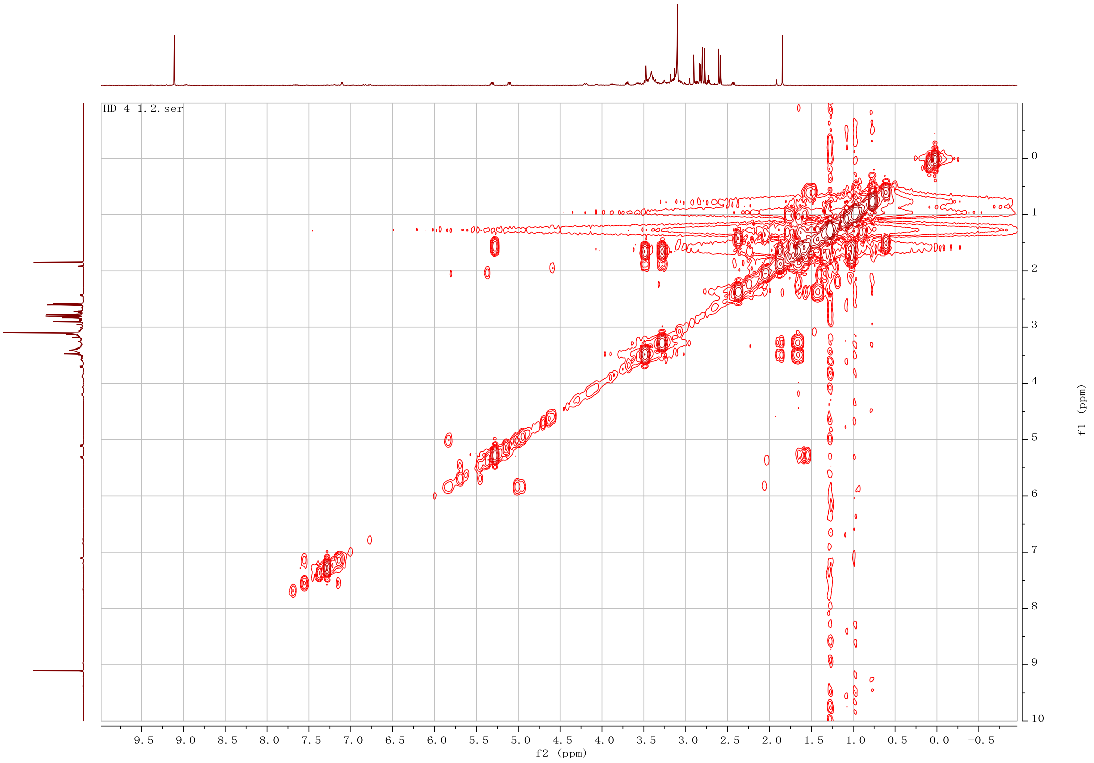

Figure S26. ^{1}H-^{1}H COSY spectrum of compound **8** in $CDCl_3$

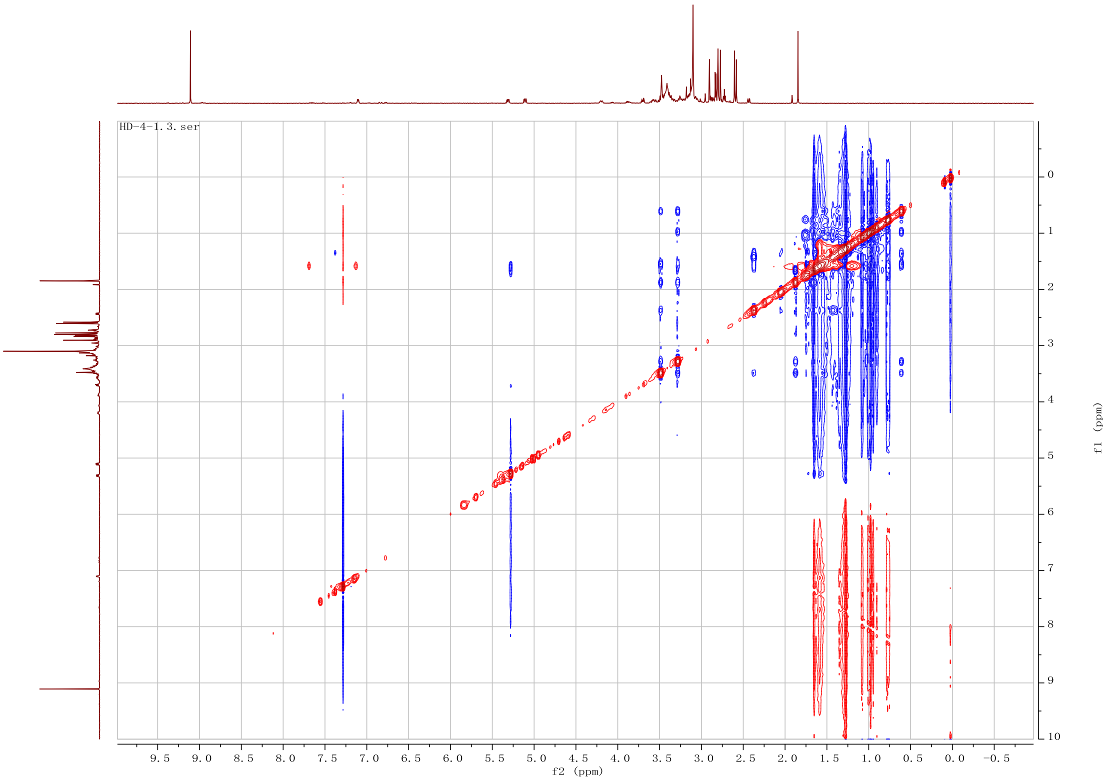

Figure S27. NOESY spectrum of compound **8** in $CDCl_3$.

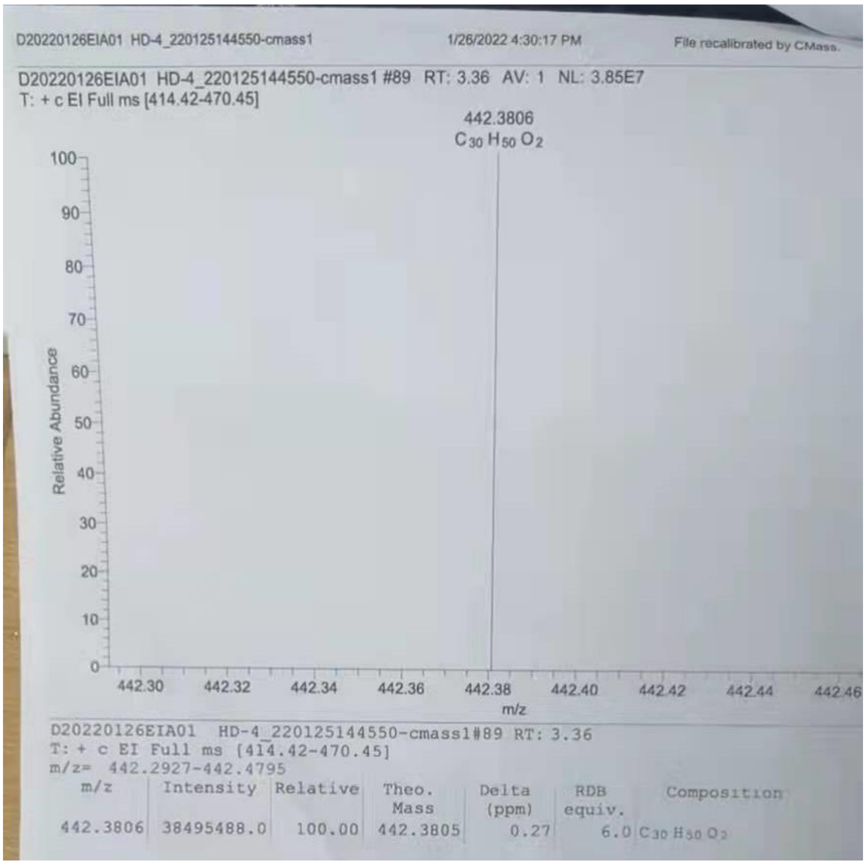

Figure S28. HRMS of compound **8** in $CDCl_3$.

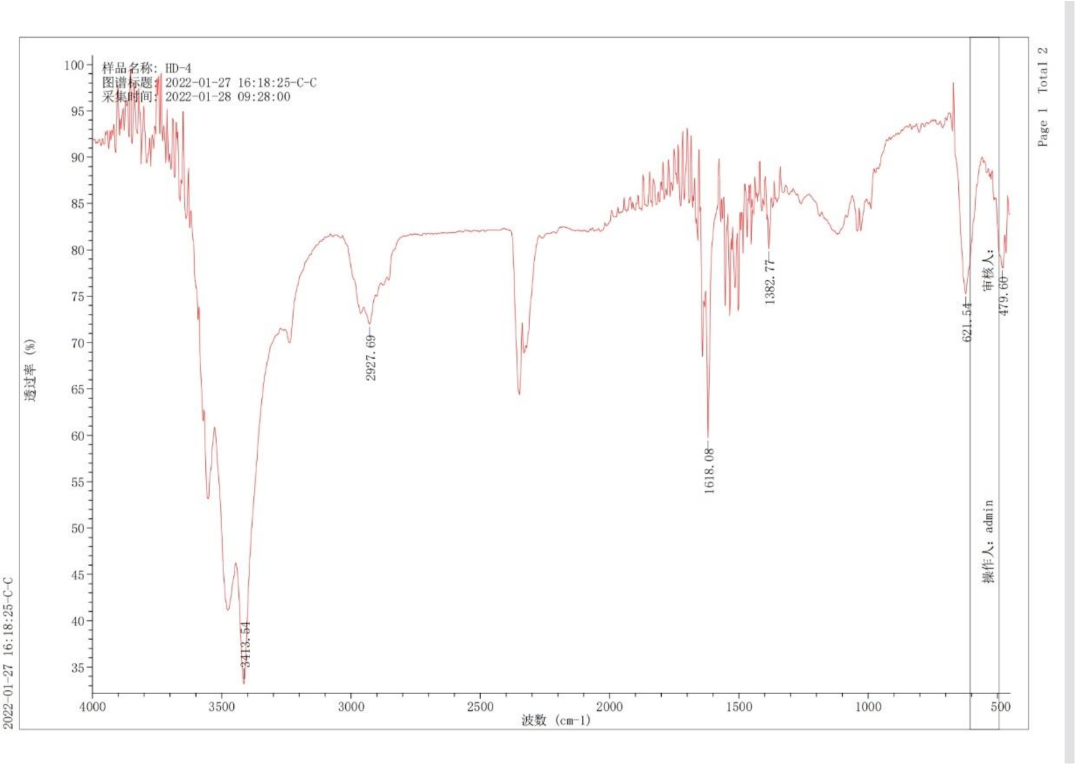

Figure S29. IR spectrum of compound **8**

波数 (cm-1)	%T
412.57	79.51
479.60	78.06
621.54	75.25
1382.77	80.13
1618.08	59.73
2927.69	72.00
3413.54	33.20

Figure S30. IR data of compound **8**

Compound **9**

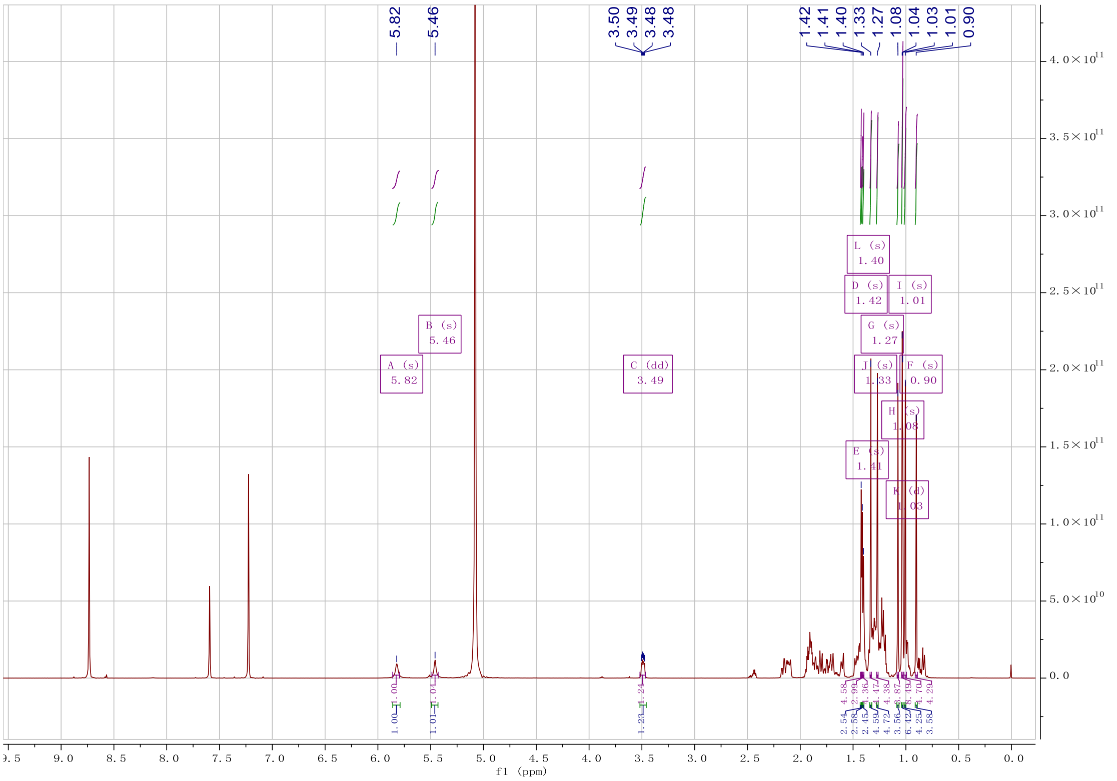

Figure S31. [1] H NMR spectrum of compound **9** in C_5D_5N (600 MHz)

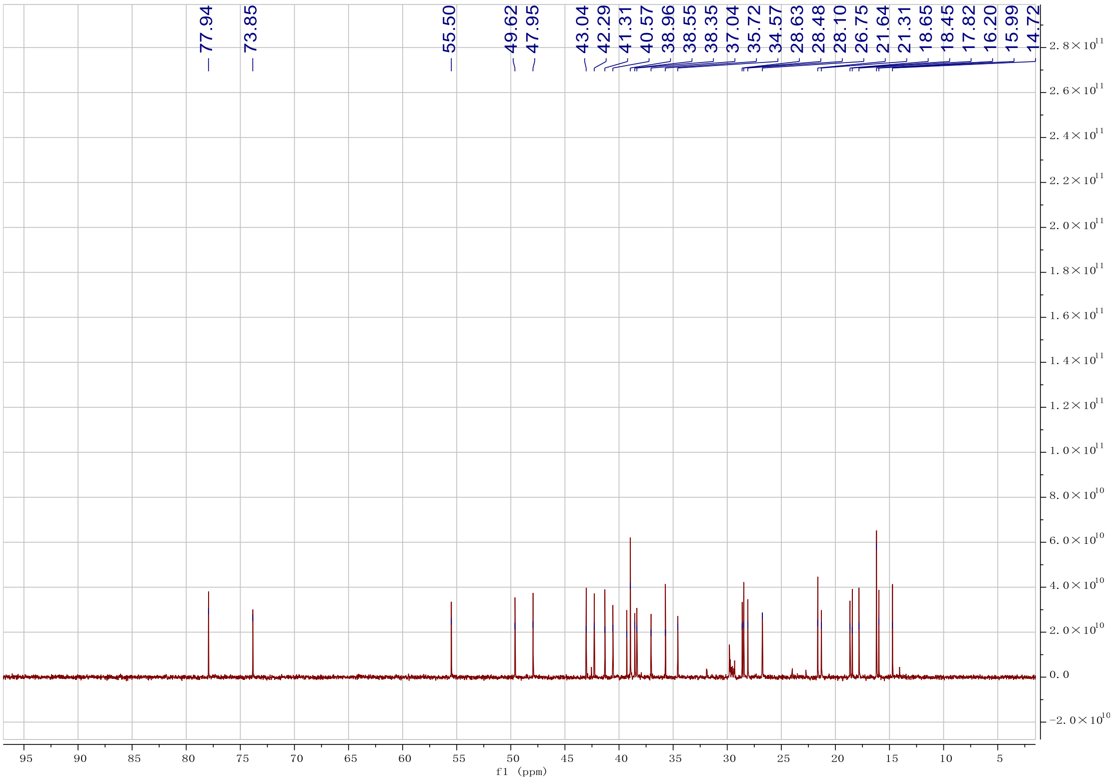

Figure S32. [13] C NMR spectrum of compound **9** in C_5D_5N (150 MHz)

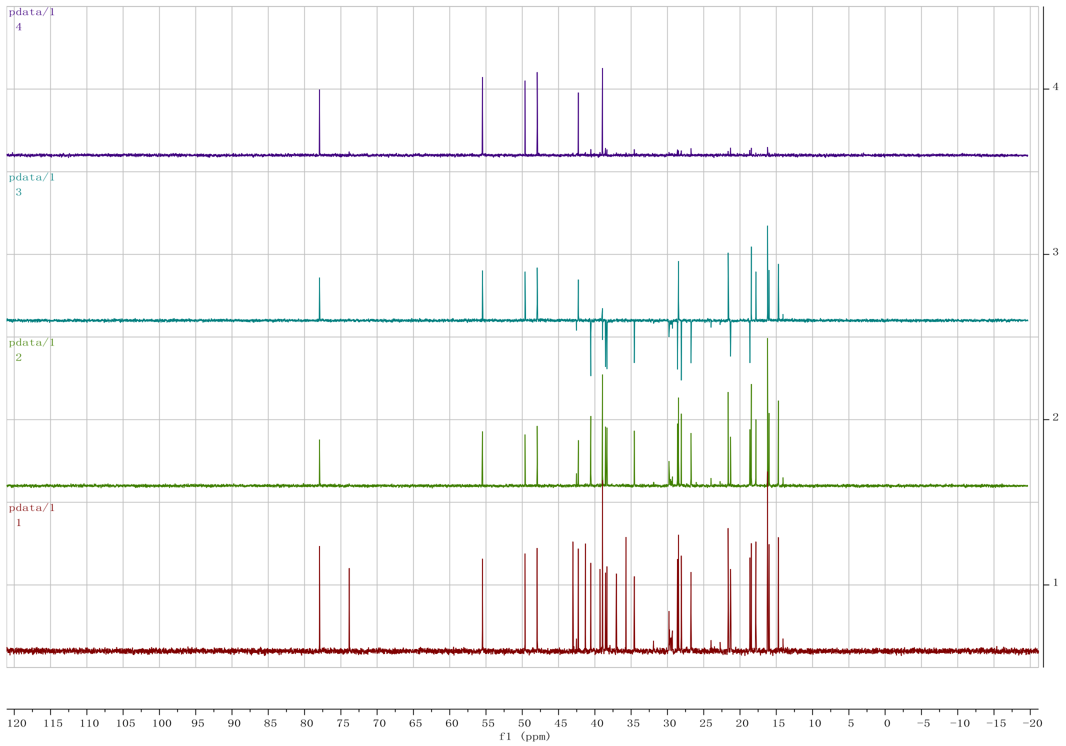

Figure S33. DEPT of compound **9** in C_5D_5N (150 MHz)

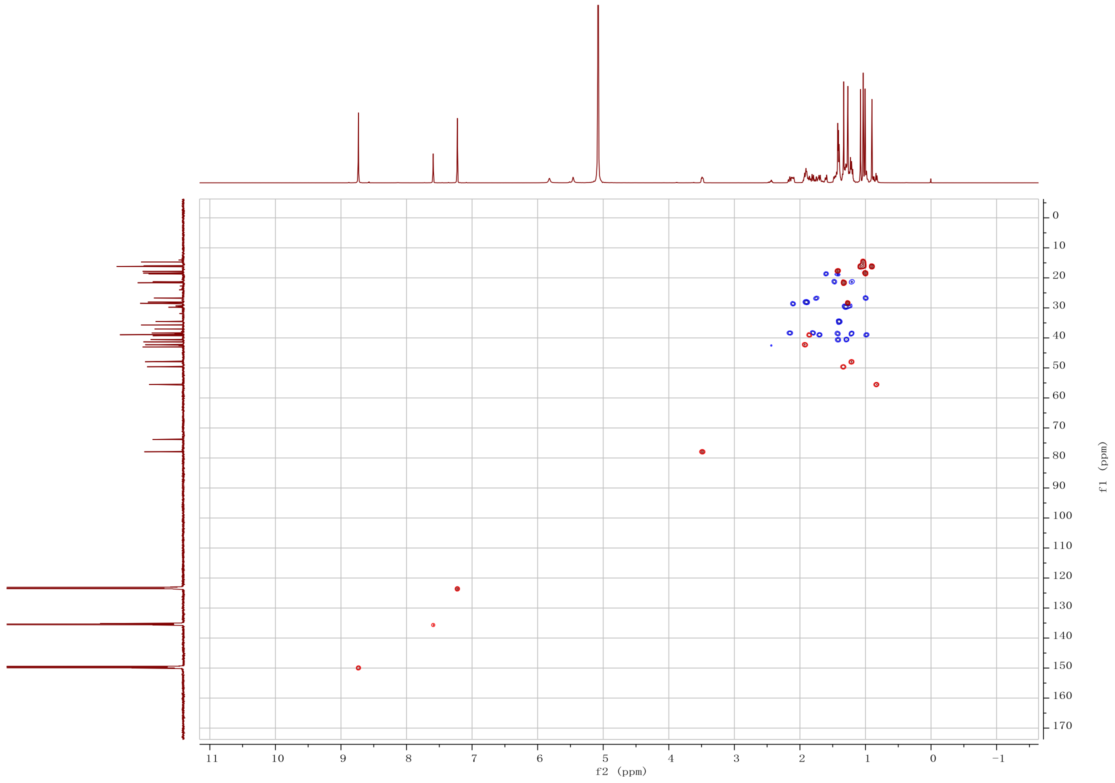

Figure S34. HSQC spectrum of compound **9** in C_5D_5N

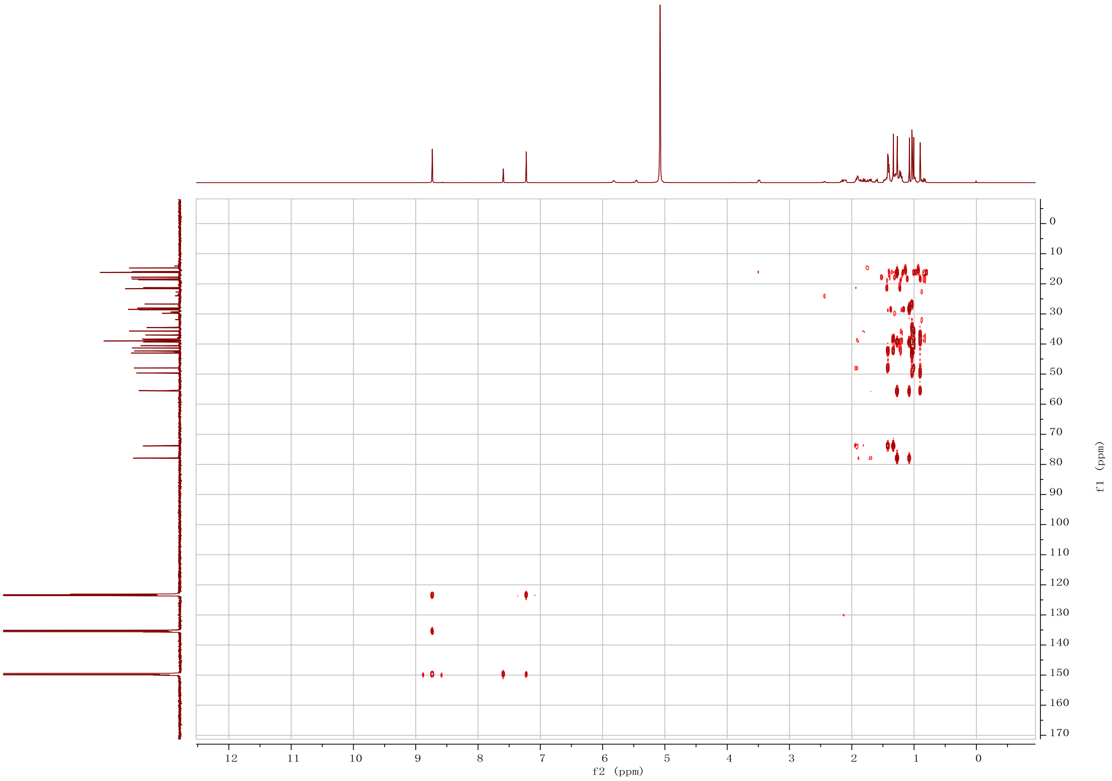

Figure S35. HMBC spectrum of compound **9** in C_5D_5N

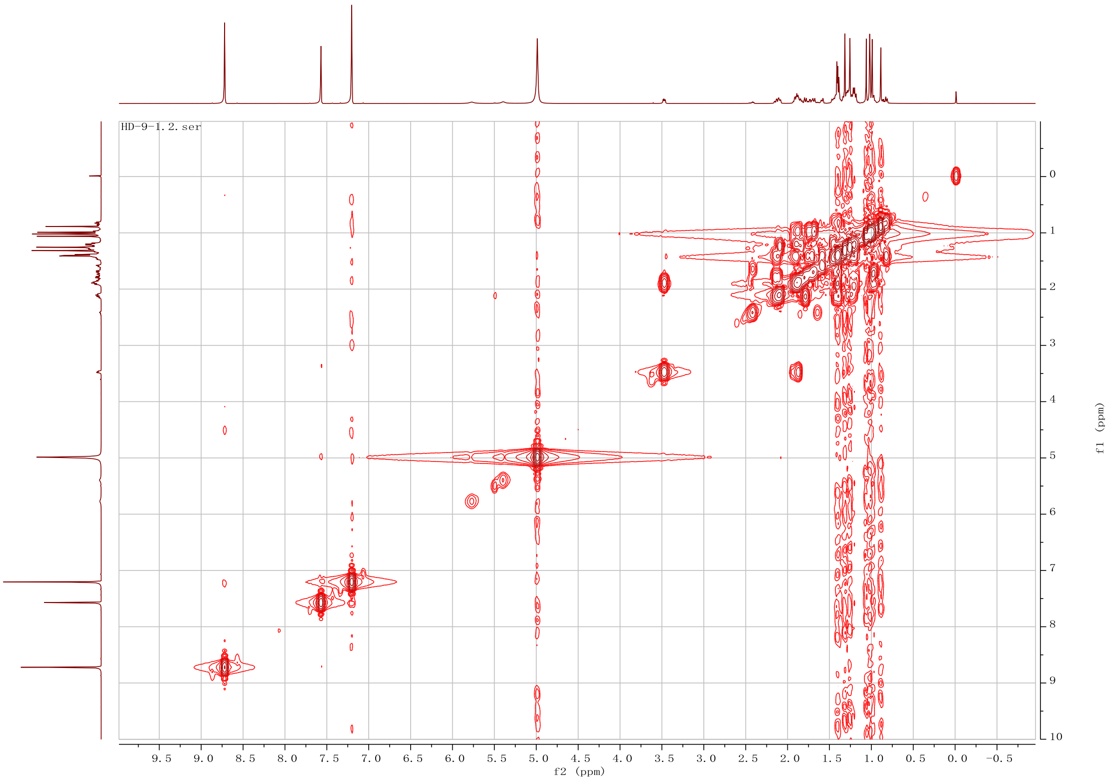

Figure S36. ^{1}H-^{1}H COSY spectrum of compound **9** in C_5D_5N

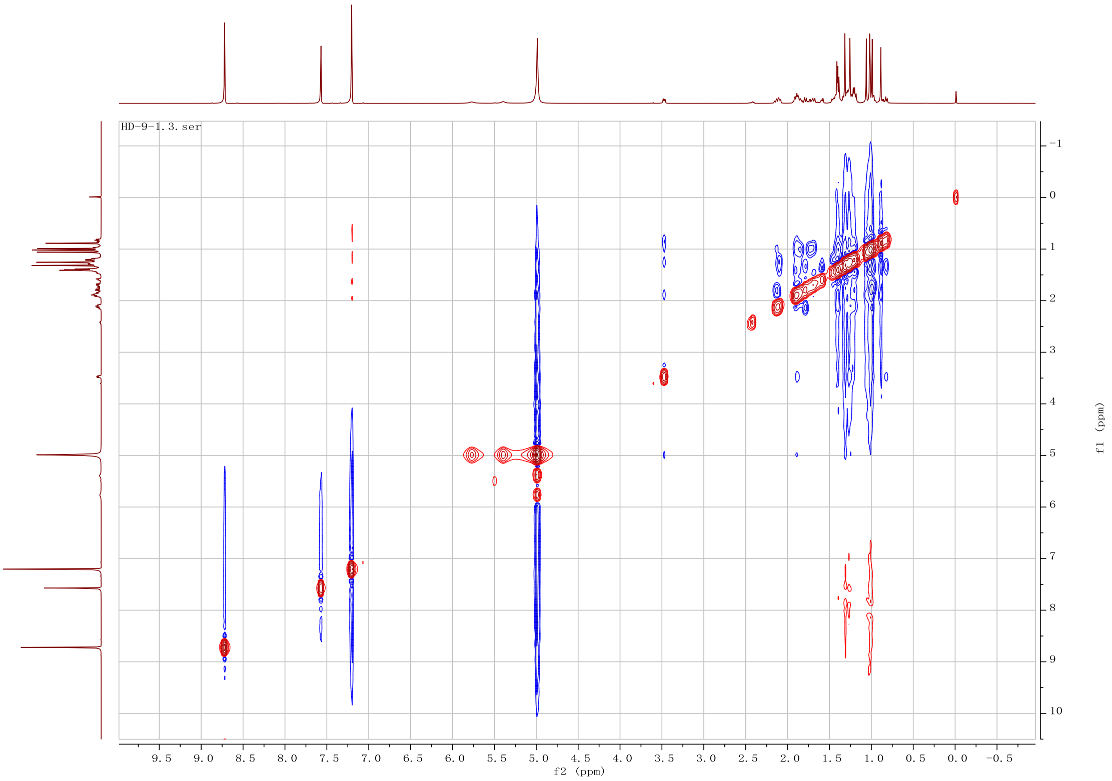

Figure S37. NOESY spectrum of compound **9** in C_5D_5N

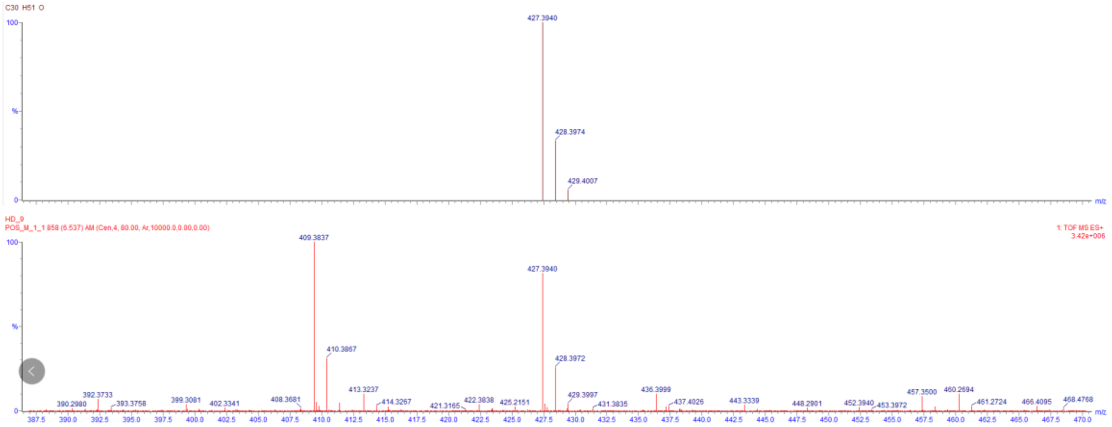

Figure S38. HRMS of compound **9** in C_5D_5N

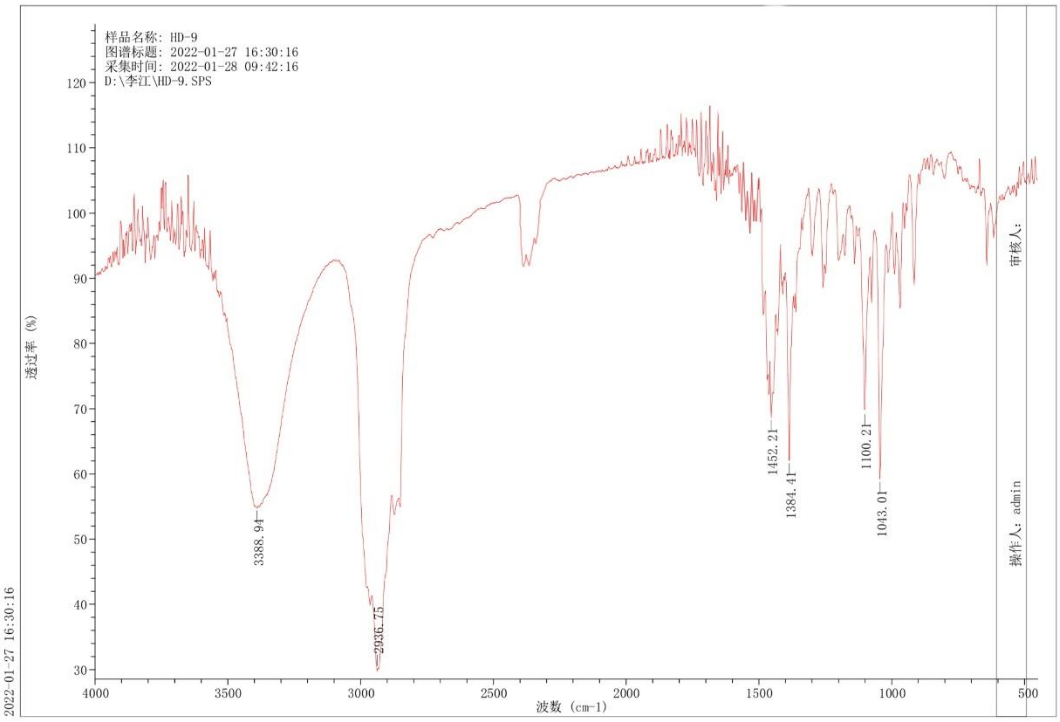

Figure S39. IR spectrum of compound **9**

波数 (cm-1)	%T
1043.01	59.21
1100.21	69.52
1384.41	62.02
1452.21	68.67
2936.75	29.82
3388.94	54.78

Figure S40. IR data of compound **9**
